# Leveraging Machine Learning and Robotic Process Automation to Identify and Convert Unstructured Colonoscopy Results Into Actionable Data: Proof-of-Concept Study

**DOI:** 10.2196/73504

**Published:** 2025-11-20

**Authors:** Elizabeth R Stevens, Jager Hartman, Paul Testa, Ajay Mansukhani, Casey Monina, Amelia Shunk, David Ranson, Yana Imberg, Ann Cote, Dinesha Prabhu, Adam Szerencsy

**Affiliations:** 1 Department of Population Health, Grossman School of Medicine, New York University, 227 E30th Street, Rm 636, New York, 10016, United States, 1 6465012558; 2 Department of Health Informatics, Medical Center Information Technology, NYU Langone Health, New York, NY, United States; 3 MCIT Clinical Systems NYU Langone, New York, NY, United States; 4 School of Medicine, Tulane University, New Orleans, LA, United States

**Keywords:** colorectal cancer, RPA, automation, colonoscopy, machine learning, artificial intelligence, robotic process automation

## Abstract

**Background:**

With rising patient volumes and a focus on quality, our health system had the objective to create a more efficient way to ensure accurate documentation of colorectal cancer (CRC) screening intervals from inbound colonoscopy reports to ensure timely follow-up. We developed an integrated end-to-end workflow solution using machine learning (ML) and robotic process automation (RPA) to extract and update electronic health record (EHR) follow-up dates from unstructured data.

**Objective:**

This study aimed to automate data extraction from external, free-text colonoscopy reports to identify and document recommended follow-up dates for CRC screening in structured EHR fields.

**Methods:**

As proof of concept, we outline the process development, validity, and implementation of an approach that integrates available tools to automate data retrieval and entry within the EHR of a large academic health system. The health system uses Epic Systems as its EHR platform, and the ML model used was trained on health system patient colonoscopy reports. This proof-of-concept process study consisted of six stages: (1) identification of gaps in documenting recommendations for follow-up CRC screening from external colonoscopy reports, (2) defining process objectives, (3) identification of technologies, (4) creation of process architecture, (5) process validation, and (6) health system–wide implementation. A chart review was performed to validate process outcomes and estimate impact.

**Results:**

We developed an automated process with 3 primary steps leveraging ML and RPA to create a fully orchestrated workflow to update CRC screening recall dates based on colonoscopy reports received from external sources. Process validity was assessed with 690 scanned colonoscopy reports. During process validation, the overall automated process achieved an accuracy of 80.7% (557/690, 95% CI 77.8%-83.7%) for correctly identifying the presence or absence of a valid follow-up date and a follow-up date false negative identification rate of 32.9% (130/395, 95% CI 29.4%-36.4%). From the organization-wide implementation to go-live until December 31, 2024, the system processed 16,563 external colonoscopy reports. Of these, 35.3% (5841/16,563) had a follow-up date meeting the relevant ML model threshold and thus were identified as ready for RPA processing.

**Conclusions:**

Implementation of an automated workflow to extract and update CRC screening follow-up dates from colonoscopy reports is feasible and has the potential to improve accuracy in patient recall while reducing documentation burden. By standardizing data ingestion, extending this approach to various unstructured data types can address deficiencies in structured EHR documentation and solve for a lack of data integration and reporting for quality measures. Automated workflows leveraging ML and RPA offer practical solutions to overcome interoperability challenges and the use of unstructured data within health care systems.

## Introduction

Colorectal cancer (CRC) is the second most common cause of cancer-related death in the United States [1] and has a growing incidence rate, particularly among younger individuals [2]. Effective CRC screening is a cornerstone of preventive health care, significantly reducing both the incidence and mortality rates associated with CRC [3]. To realize the full benefit of CRC screening, an accurate and timely recall of patients for follow-up screening is needed.

Despite the availability of electronic health record (EHR) tools to facilitate reminders for CRC screening follow-up, these tools often fail to address clinician needs [4,5]. The limited interoperability of health information exchanges between health care systems and reliance on unstructured data to convey recommendations for follow-up place a high documentation burden on clinicians, leading to substantial missed opportunities for improved CRC follow-up screening and surveillance [6-8].

EHR tools are rarely responsive to patient-specific factors or colonoscopy and pathology findings. Consequently, recalling a patient for more frequent follow-up screening requires a manual update [9]. To enhance CRC screening follow-up accuracy, while minimizing documentation burden, solutions that automate the retrieval and processing of colonoscopy results are needed. An automated process for updating CRC screening follow-up–based colonoscopy findings is likely to increase the number of patients with a documented guideline-concordant CRC follow-up screening interval [10,11].

As with nearly 80% of EHR data [12], colonoscopy and pathology findings are frequently documented within the EHR as free text [13]. Further complexity arises when colonoscopy results are received as inbound medical records from external sources. These records, often received via fax, mail, or email, require additional steps to document the relevant data into the EHR, posing a significant administrative burden for documenting CRC screening due dates [14].

Natural language processing (NLP) models have the potential to address barriers associated with leveraging unstructured data and accurately extract relevant data from free-text colonoscopy and pathology reports [15]. However, NLP alone cannot address all workflow steps required to convert text-based reports into structured fields in the EHR. It is essential to pair the model with supplemental tools for data entry. A solution is needed that orchestrates multiple technologies to address each step in the workflow necessary to update the appropriate CRC follow-up dates.

With rising patient volumes and a focus on quality, our health system needed a more efficient way to ensure accurate documentation of CRC screening follow-up intervals from inbound colonoscopy reports. To address this, we developed an integrated workflow using machine learning (ML) and robotic process automation (RPA) to extract and update follow-up dates from unstructured data. With the objective of demonstrating a proof of concept, in this paper, we outline the process and validity of an approach to process inbound colonoscopy reports that integrates available tools to automate data retrieval and entry within the EHR. We hypothesize that this framework can be adapted to other clinical information to improve reporting, data accuracy, and workflow efficiency.

## Methods

### Overview

This proof-of-concept study was part of an institutional initiative to improve the accuracy of documented CRC screening follow-up recommendations extracted from unstructured colonoscopy reports from external organizations. The study developed an integrated workflow using existing technologies to address this care gap and assessed its validity. This study consisted of six stages: (1) identification of gaps in operationalizing recommendations for follow-up CRC screening from external colonoscopy reports, (2) defining process objectives, (3) identification of available technological tools, (4) creation of process architecture, (5) process validation and refinement, and (6) health system–wide process implementation.

### Study Setting and Participants

The New York University Langone Health (NYULH) system is a private, nonprofit hospital system serving the greater New York area and Florida with over 9 million patients. The NYULH system uses Epic Systems as its EHR platform. For the process validation component of this study, EHR data were extracted prospectively for a series of 4 sequential simple random samples of scanned colonoscopy reports from adult patients aged 45 to 75 years from external sources between August 2023 and September 2023.

### Health Informatics Care Gap

Ensuring timely follow-up for CRC screening was identified as an important quality measure for the health system. Accurately tracking patients due for CRC screening is facilitated by having the next colonoscopy follow-up date documented discretely in the EHR’s health maintenance (HM) activity.

Gaps in this process frequently occur when colonoscopy reports from external organizations are scanned into the health system’s EHR. Since these documents contain unstructured data, critical information remains inaccessible unless manually transcribed into the EHR [12,13]. If this information is not updated manually, the HM follow-up screening interval defaults to 10 years, which may not be appropriate for many patients [8]. As a result, the HM activity often fails to reflect the recommended follow-up intervals indicated in the colonoscopy report unless users manually update it. Factors such as procedural findings, pathology results, and patient-specific risk factors may necessitate more frequent surveillance. To ensure transparency and awareness for both patients and clinicians, as well as for accurate reporting, this information should be documented discretely in the appropriate fields within the EHR.

Manually updating the HM follow-up intervals is time-consuming, often leading to unchanged follow-up dates in the EHR and failing to reflect the appropriate recall time frame. This documentation gap can result in missed or delayed surveillance, potentially impacting patient outcomes. To address this challenge, an efficient and effective process is needed to extract and process unstructured text from colonoscopy reports to automatically update HM follow-up intervals.

### Defining the Process Objective

After identifying the care gap, the objective was to develop a process to automate updating the HM based on the external colonoscopy reports. We sought to leverage ML to identify follow-up recommendations within the reports and RPA to automate the entry of recommended recall dates into the HM activity.

It was also determined that the process must operate within the following constraints: (1) use existing technologies within the health system, (2) not override any recommendations that have been manually entered by a clinician, (3) ML engine identifies follow-up date above a technical performance threshold of >70% confidence; and (4) No human intervention after the colonoscopy report has been scanned into the EHR.

Although pathology results should ideally serve as the gold standard for assessing follow-up intervals, due to limited availability, the recommendation listed within the colonoscopy report was deemed a sufficiently accurate alternative for the purpose of updating HM.

### Orchestrated Workflow Development

To develop the orchestrated workflow, the team first broke the process into concrete steps. We then mapped out existing technological components within the healthy IT ecosystem needed for each action. The steps included: (1) daily extraction of scanned external colonoscopy reports, (2) a mechanism for an ML model to receive and analyze those reports to identify the next follow-up date, and (3) an automated process that updates the HM activity when appropriate.

The team established care assumptions to guide when the process updates would take precedence over existing HM follow-up frequencies. These assumptions included: (1) all updates must be associated with a colonoscopy order; (2) the bot should only update HM if the scanned report is more recent than the last documented colonoscopy; (3) the scanned report would not override manual entry; (4) the bot should not update a follow-up date if the patient has an existing earlier follow-up date; (5) if the ML engine’s determination of follow-up date is below the performance threshold, the report should be queued for manual review; (6) if an outstanding colonoscopy order exists and the procedure date is after the order date, the report should be scanned to that order; and (7) if there is a range of follow-up dates (eg, return in 3‐5 y) the bot will use the earlier date. Based on these assumptions, business exceptions within the decision rules were defined to establish scenarios in which it would be inappropriate for the bot to update the HM.

### Identified Technological Tools

#### Overview

Existing technologies were evaluated for their applicability to each workflow step, resulting in a process comprising six key components, including: (1) an internally developed ML model, (2) fax management software, (3) a document management platform, (4) input spreadsheets, (5) RPA, and (6) Epic’s HM activity.

#### Fax Management Software 

RightFax (OpenText) is an enterprise fax server software that allows the organization to send and receive faxes. It also transforms faxes for easier management and integration into other digital systems, including the EHR [16]. RightFax was used to receive colonoscopy reports from external sources.

#### ML Techniques

ML techniques, including optical character recognition and NLP, enable systems to “read” documents and extract relevant information [17,18]. ML is a subset of artificial intelligence (AI) that enables systems to learn patterns from data and make predictions or classifications without explicit programming. These classifier algorithms can identify patterns, structures, and key data within various document formats, automating the extraction process and reducing the need for manual intervention. This capability is widely used in data entry, document analysis, and information retrieval. A proprietary model was internally developed to identify the follow-up dates for the next screening. The model was trained on 7021 unique documents, 90.6% (6358/7021) of which were colonoscopy reports, 6.7% (473/7021) were pathology reports, and 2.7% (190/7021) were other types of documents. Details of the ML model training data set can be found in Multimedia Appendix 1. This process required model training by a clinical subject matter expert.

The ML was validated using a 4:1 patient-based split with manual review of a sample of 1758 documents and associated RPA actions in the EHR. The validation documents included 90.8% (1597/1758) colonoscopy reports, 6.5% (114/1758) pathology reports, and 2.7% (47/1758) other types of documents. Details of the ML model validation data set can be found in Multimedia Appendix 1.

#### RPA Technology

UiPath RPA is a technology that uses software robots, or “bots,” to automate repetitive and rule-based tasks typically performed by humans. These RPA bots can work across platforms and mimic human actions such as data entry, processing transactions, and managing records, thereby increasing efficiency and reducing errors. Unlike AI, RPA follows predefined workflows and does not learn or adapt on its own [19]. In this case, the RPA tool “read” the output of the ML engine, updated the HM activity date if applicable, and documented the actions taken. If any business exceptions existed, they were similarly documented. Finally, the RPA sent a summary of the completed process to operational leadership for review.

#### Document Management Platform

OnBase is an enterprise content management platform developed by Hyland Software. It helps organizations manage documents in a unified system, acting as a digital filing cabinet with advanced automation capabilities. OnBase supports document storage, quick retrieval, and task automation, enhancing efficiency and compliance [20]. In this process, OnBase is used as the underlying application where the reports were scanned into the EHR. A specific directory was created to process incoming colonoscopy reports so that they can be processed by the ML model.

#### Input Spreadsheet

The input spreadsheet was a Microsoft Excel document that was used as a working tool to list the findings and follow-up dates of the colonoscopy reports as identified by the ML model. Any findings with low confidence were indicated as such. The RPA solution reviewed the spreadsheet, “read” the follow-up dates, and documented what actions it took.

#### HM EHR Feature

Epic Systems’ HM activity is an EHR feature designed to help providers manage and track preventive care and chronic disease management for patients. It allows health care organizations to set up and monitor recommended screenings, vaccinations, and follow-up care based on clinical guidelines, patient demographics, and medical history. It serves as a designated location in the EHR to document completed colonoscopies and the recommended follow-up date. However, it is not linked to clinical notes and must be manually updated to reflect changes due to pathology findings or patient-specific conditions [6].

### Process Validation

#### Overview

Prior to systemwide activation, outcome validation was performed via manual review of a sample of colonoscopy reports and associated RPA actions in the EHR. The measures of outcome validity were the number of reports with a missed follow-up date, and the number of reports with a correctly updated HM. Each colonoscopy report was reviewed by trained clinical analysts (DR and CM) for accuracy. Discrepancies were reviewed with the physician informaticist lead (AS). The validation outcomes were divided into two phases: (1) ML evaluation, determining whether a follow-up date was identified within the colonoscopy report and ready for bot processing; and (2) RPA bot action documented in the EHR. Reports not ready for the bot were categorized as (1) correct, with no follow-up date specified; (2) incorrect, having a follow-up date listed that was not picked up by the ML; or (3) an inaccurate follow-up date was identified. Outcomes for reports processed by the RPA update were categorized as: (1) a correct HM update; (2) correct business exception assignment; or (3) incorrect HM update. Four rounds of validation testing were performed with a prospective sample of 690 reports manually reviewed until a saturation of automated outcomes was observed.

#### Process Refinement

A protocol was established to modify and refine the ML algorithm and bot activities if error patterns were identified during validation. After each round of validation testing, if an error was addressable, the team adjusted either the ML model or RPA based on the error source.

#### Governance Approval

Validation study findings were presented at leadership committee meetings to obtain governance approval for system-wide implementation. Besides confirming the process’s validity, the approval process emphasized potential improvements in HM follow-up date accuracy compared to the current reliance on a default 10-year interval and limited manual updates by clinicians.

### Process Implementation

After validation, refinement, and all necessary approvals, the process was implemented in the live environment. The implementation was performed for received documents labeled as colonoscopy reports only, not all document types used in the training and validation of the ML model. This began with a period of careful monitoring of the process known as hypercare [21]. The automated process was first implemented in the live environment as small batches, where 10 reports were run through the process and assessed for validity before proceeding to the next 10. This procedure was performed until it was determined that the process performed appropriately in the live environment. After the initial phase, the process was expanded to analyze all prospectively received external colonoscopy reports.

### Ethical Considerations

As an operational quality improvement project, the NYULH institutional review board waived the need for approval for the study procedures. A waiver of Health Insurance Portability and Accountability Act (HIPAA) authorization and consent was included to access patient medical records. All study procedures complied with institutional ethical and privacy standards. Informed consent was waived and no compensation was provided.

## Results

### Overview

**Figure 1.**
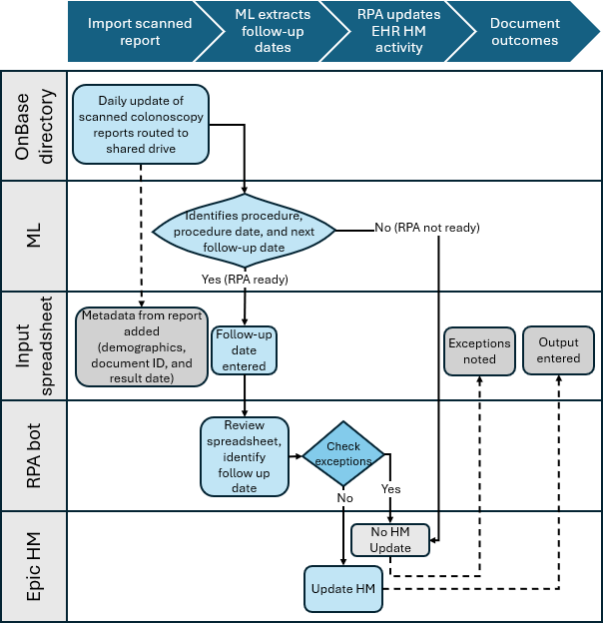
Automation process and technology tools used in each process step for the automation proof of concept to identify and convert unstructured colonoscopy results into actionable data note: HM: health maintenance; ML: machine learning; RPA: robotic process automation;

Our system established a streamlined, 3-step process integrating preexisting technologies to automate updates to the CRC screening follow-up date based on external colonoscopy results. The process steps, the workflow, and technology resources used for each function are presented in Figure 1.

The three steps are as follows:

1. Documentation identification and routing: scanned colonoscopy reports are identified and sent to a designated work queue in the OnBase shared drive.
2. ML processing: an ML model verifies that the document is a colonoscopy report, then extracts key data, including patient ID, report date, and follow-up recommendations. If data meets predefined accuracy thresholds, it is added to an input spreadsheet and labeled “RPA ready.” If below thresholds, the document is marked “RPA not ready” and sent to a work queue for human validation. ML model details, including model parameters, training processes, and performance are reported in Multimedia Appendix 1.
3. RPA execution: the bot processes “RPA ready” entries by accessing the patient’s EHR, comparing the extracted follow-up date with existing HM follow-up frequency, and updating the recall date if no business exceptions are detected. All processed data, including business exceptions, are documented in the input spreadsheet.

### Business Exceptions

Nine business exceptions were defined such that if any criteria were met, the bot would not update HM. These criteria included: (1) the follow-up date in the scanned document is longer than existing HM follow-up frequency, (2) a patient has a HM frequency modifier that sets a patient-specific screening frequency, (3) the next HM due date has been manually assigned after the scanned procedure date and these exceptions ensure the bot never takes precedence over a provider-set screening frequency or patient-specific factors, (4) the report has a follow-up date over 10 years, (5) the input spreadsheet cannot assess follow-up date, (6) the procedure date linked to that document does not match the scanned document date, (7) the patient does not have the colonoscopy screening topic assigned in Epic due to age criteria, (8) the patient is not found, or (9) an order for the scanned colonoscopy report is not found. This exception ensures that the HM updates are associated with an appropriate colonoscopy order.

### Process Validation Outcomes

**Figure 2.**
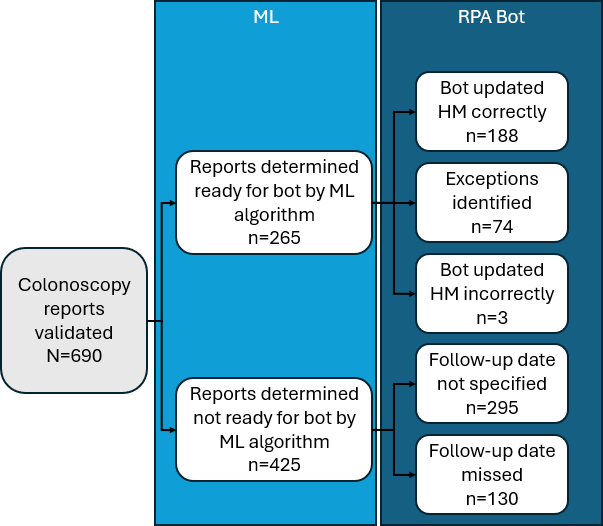
Process validation outcomes for the automation proof of concept used to identify and convert unstructured colonoscopy results into actionable data note: HM: health maintenance; ML: machine learning; RPA: robotic process automation.

Figure 2 presents the validation outcome findings. Process validity was assessed for 690 scanned colonoscopy reports. Of these, 61.6% (425/690) were categorized by the ML algorithm as not ready for the bot. Of those, 30.6% (130/425) listed a follow-up date not detected by the ML algorithm and 69.4% (295/425) did not specify a follow-up date. Among the reports identified as ready for the bot, 70.9% (188/265) HM were correctly updated, 27.9% (74/265) were flagged with an update exception, and 1% (3/265) HM were incorrectly updated. During the process validation, the final document processing overall automated process had an accuracy of 80.7% (557/690, 95% CI 77.8%-83.7%) for correctly identifying the presence or absence of a valid follow-up date (a measure that requires both the identification of the follow-up interval and the result date), determining the absence of a follow-up date, or recognizing a business exception that precluded updating the follow-up date. The process demonstrated a false negative identification rate of 32.9% (130/395, 95% CI 29.4%-36.4%) for follow-up date identification. Based on validation data, we estimate that this process correctly identified and updated 27.2% (188/690) of cases, either correcting instances that would have otherwise defaulted to an incorrect interval or confirming a 10-year follow-up interval was appropriate. Validation outcomes for the ML model specifically can be found in Multimedia Appendix 1.

### Process Refinements

During validation, the three incorrectly updated HM were due to (1) the bot creating an update based on an older report and (2) overriding a provider modification. These errors led to process refinement by updating the RPA steps to validate clinical information. The process error rate of 32.9% (130/395, 95% CI 29.4%-36.4%) for missing an available follow-up date was deemed acceptable, as refining the model to be more sensitive led to the extraction of incorrect dates, seen as a greater issue than missing a date.

### System-Wide Implementation

The solution was implemented system-wide on October 4, 2023. From go-live until December 31, 2024, the system processed 16,563 external colonoscopy reports. Of these, 35.3% (5841/16,563) had a follow-up date meeting the ML model threshold and thus identified as “RPA Ready.” Of the “RPA Ready” documents, the HM topic was successfully updated in 77.2% (4512/5841) of patient records. Among all processed reports, 72.8% (12,051/16,563) were not updated; this included 22.8% (1329/5841) that were “RPA Ready,” but had business exceptions. Of the “RPA Ready” documents, 3.9% (225/5841) had a patient-specific follow-up date modifier, 3.2% (189/5841) had a more recent procedure, 6.8% (398/5841) were manually updated by a provider, 8.3% (485/5841) were outside standard screening age and did not have a colonoscopy HM topic assigned, and 0.6% (32/5841) had a discrepancy where the procedure result date did not match the scanned document.

## Discussion

### Principal Findings

This proof of concept demonstrated the feasibility of using ML and RPA to develop an automated process capable of extracting key clinical information from unstructured documents and converting it into discrete, accessible data. The system improved the accuracy of CRC screening follow-up dates while reducing the documentation burden on clinical staff. This pragmatic and innovative approach represents one of the first end-to-end solutions to address the challenges of processing faxed, text-based results by converting free text into structured data. This work provides a template for health care systems to reduce documentation burden, improve follow-up date accuracy, and address care gaps arising from a lack of system integration.

The process automates all workflow stages from the initial receipt of the free-text report to the entry of the follow-up date into the EHR. The use of ML combined with RPA allows for the process to function across workflow stages and software that would not be possible without human intervention if using existing NLP- and rule-based methods alone. Traditionally, this information would be difficult to locate within existing workflows, limiting its influence on patient recall processes. By structuring and integrating this data, the system ensures timely follow-ups and supports downstream workflows to recall patients within recommended timeframes, ultimately improving preventive care management.

Many important data elements are not updated in the EHR because updating them is time-consuming [11]. This strategy reduces documentation burden while minimizing data entry errors. As demonstrated in the process validation study, this process improved the accuracy of HM CRC screening dates by nearly 30% by updating records that would have otherwise remained unchanged. This has the potential to substantially impact downstream patient outcomes. The process was designed to err on the side of being clinically conservative (eg, selecting earlier recall dates if a range was present in the colonoscopy report). While selecting the earlier date in the recall range is a clinical guideline-concordant decision, this conservative approach may increase the rate of colonoscopy compared to selecting the mid- or end-range recall date [22]. During process validation, the accuracy for finding a valid follow-up date was slightly lower than the recall found in the ML model testing (Multimedia Appendix 1). This was anticipated, as a follow-up date is a measure that requires both the identification of the follow-up interval (recall 99.9%) and the result date (recall 86.7%) as well as the recall of other document features; however, this decline in performance from the ML model to the complete process should be considered during process development. Future research should examine whether the increase in screening date accuracy and conservative follow-up date selection translates into improved CRC clinical outcomes.

Beyond improving follow-up date accuracy, this workflow reduces the administrative burden on clinical staff, ensuring timely and appropriate care. By automating the update of recall dates for nearly 30% of scanned colonoscopy reports, health systems can save significant labor time. Even if each report requires just a few minutes of manual processing, the time savings can add up to hundreds of hours—especially in large health systems like NYULH, which may order upwards of 80,000 colonoscopies annually. Transforming unstructured text into structured data also has significant implications for quality reporting. By automating data extraction and integration into the EHR, health care systems can more effectively track completed screenings and tests, ensuring comprehensive reporting on quality measures, closing care gaps, and improving population health management.

### Comparison With Prior Work

The use of automation, including RPA, has previously been proposed as a theoretical solution to address limitations in the EHR and other elements of data-driven health care [23-27]. However, prior literature has not presented a fully integrated end-to-end automated workflow. Prior research has primarily focused on data automation in structured formats [27], such as health care claims [25,26] and the extraction and processing of EHR data for research [27-32]. A few studies present cases for using ML and AI for other data types [30,32], but their full integration into health system operations remains hypothetical. Our work builds upon previous research to demonstrate the feasibility and validity of implementing a pragmatic, automated workflow within the EHR to address real-world challenges. While we developed a proprietary model to extract follow-up dates from colonoscopy reports, we anticipate advancements in generative AI will enable multimodal large language models to achieve similar outcomes. However, validation testing is needed to determine large language model accuracy in these use cases. These models can support this use case and be scaled to extract other critical clinical data from unstructured text, reducing documentation burden and enhancing automation in health care systems.

This process framework can address inefficiencies from the lack of EHR interoperability or discordant information within various areas of the EHR. Despite the progress of EHR interoperability, health care organizations still rely on electronic fax transmissions of clinical information containing important yet unstructured data. Our institution has adapted the described process to automate the documentation of follow-up dates for internally performed colonoscopies documented within ancillary systems and interfaced back as free text reports. Before a recent 2023 institutional effort to code colonoscopy pathology findings [11], all colonoscopy reports were stored as unstructured text. Using a modified version of this automated process, the institution has retrospectively processed 80,864 internal reports from May 2020 to April 2023, updating 71.8% (58,060) CRC screening HM follow-up dates. This successful application of ML combined with RPA highlights their potential to address other high-burden, low-complexity tasks in the EHR and could address deficiencies in structured EHR documentation. By standardizing data ingestion, extending this approach to various unstructured data types can address deficiencies in structured EHR documentation for tests performed at outside organizations and solve for a lack of data integration and reporting for quality measures. Moreover, scaling similar processes to other report types with discrete values, such as cholesterol or HbA_1c_, may demonstrate an even greater success.

Incorporating ML into the workflow was critical to the automated process’s utility. Nearly 80% of EHR data [12], including colonoscopy reports and pathology findings, are documented as free text [13]. Therefore, the critical information within the reports remains inaccessible in the EHR unless manually transcribed [12,13]. Most proposed automations cannot use free-text data and rely on discretely stored data, such as laboratory result values [27], which is unavailable for many EHR data sources unless data entry systems and clinician behaviors are modified. By documenting discrete data without requiring behavior change or system structure modifications, these tools may facilitate broader data capture automation. As AI advances, integrating more sophisticated large language models may further refine and scale these efforts. Future work should evaluate the long-term impact, scalability across different health care systems, and additional opportunities for automation to support clinical workflows, quality reporting, and patient care.

### Limitations

This study had several limitations. First, as this workflow integrates multiple disparate technologies to create an automated process, it is susceptible to technical issues from changes to any of the individual components. For example, system updates can change the user interface, causing the bot to no longer recognize its inputs. To address this, the process requires consistent monitoring and maintenance that may incur long-term maintenance costs.

Second, using colonoscopy reports rather than pathology results as the source for follow-up interval means some dates may require updating after pathology results are available. However, as colonoscopy reports are more commonly transmitted from external sources, this decision was pragmatic and provided a sufficiently accurate representation to process the greatest number of results. Future iterations may consider incorporating logic based on the pathology results if included along with the colonoscopy report.

In addition, the ML model used as a component of this intelligent document processing pipeline was not validated on an external dataset and was only validated within 1 health system. While training and internal validation included multiple data sources (ie, Cologuard and colonoscopy, pathology results, documents received from various external clinics), validation in only 1 health system may limit the generalizability of this tool, and adaptation may be needed for other health care environments, particularly those with different EHR systems. While efforts were iteratively made to improve the model as more types of report formats are received, regular refinements including larger data training sets are needed to increase model recall. However, due to finite resources and available backup manual processes, the accuracy was deemed sufficient for operational purposes and our model is not regularly refined. The percentage of follow-up dates updated by the process, however, was lower than would be expected based solely on estimated model performance. While approximately 31% (130/425) of not identified follow-up dates were due to the ML model’s technical limitations, the limited update rate was primarily due to clinical factors as well as purposeful operational decision-making. Clinically, the process’s effectiveness was limited by the high rate of missing elements in the received colonoscopy reports [33,34]. From an operational perspective, this process was likely to remain largely unsupervised once deployed. While automation outperforms existing human-only workflows for updating HM, the automation of clinical documentation can pose ethical and safety concerns if errors occur. To ensure accountability and trust, governance safeguards are essential, and taking a more cautious approach is advisable when designing automated systems intended to fully replace manual oversight. To mitigate potential risks, we institute a number of business exceptions designed to function as safeguards, thereby erring on the side of caution. These business exceptions included limiting overwrites of prior clinician input, allowing clinician overrides, reviewing records with high uncertainty, and clearly assigning responsibility to prevent overreliance on automation. Therefore, a substantial number of records were not updated by the automation process due to available prior clinician input, even if the model correctly identified a follow-up date. If no clinician actions were taken prior to the process being executed, the rate of update would be expected to be higher than what was seen in a real clinical setting.

### Conclusions

This study demonstrates the feasibility of developing a high-validity, automated process to update CRC screening follow-up recommendations within the EHR using scanned colonoscopy reports. By leveraging ML and RPA, we successfully extracted key clinical information from unstructured documents and converted it into structured, actionable data. The success of this approach highlights the potential for similar automation strategies in other high-volume, low-complexity documentation tasks in healthcare, enhancing efficiency, reducing manual errors, and improving patient outcomes.

## Supplementary material

10.2196/73504 Multimedia Appendix 1 Technical Summary of natural language processing (NLP) model development for colonoscopy report extraction.

## Abbreviations

CRC: colorectal cancer
EHR: electronic health record
HIPAA: Health Insurance Portability and Accountability Act
HM: health maintenance
ML: machine learning
NLP: natural language processing
NYULH: New York University Langone Health
RPA: robotic process automation
