# Supplementary material for: Leveraging Machine Learning and Robotic Process Automation to Identify and Convert Unstructured Colonoscopy Results Into Actionable Data: Proof-of-Concept Study
Source: JMIR Med Inform. 2025 Nov 20;13:e73504. doi: 10.2196/73504 (PMC12634012; doi:10.2196/73504)
Supplement: Multimedia Appendix 1 [file medinform-v13-e73504-s001.pdf]

Appendix 1:
Technical Summary of NLP Model Development for Colonoscopy Report Extraction

This appendix provides an overview of the development and validation of a machine learning model designed to extract structured information from scanned colonoscopy reports using natural language processing (NLP). The machine learning (ML) model was developed to support automation of data entry into patient records.

1. Model Development Overview

The model processes scanned colonoscopy reports extracted weekly from OnBase, filtered by document type. These documents are first converted to raw text using AWS Textract, an optical character recognition (OCR) tool that has been successfully applied in biomedical contexts to extract structured data from unstructured medical documents [1][2].

Input Format:
- Scanned image files of colonoscopy-related reports
- Linked patient/order metadata from OnBase

Output Format:
- Excel spreadsheet containing extracted entities and a readiness score for robotic process automation (RPA)

2. Development Process

The model was designed to extract the following entities:

- First Name (FN)
- Last Name (LN)
- Date of Birth (DOB)
- Follow-Up Interval (FOLUP)
- Order Date (ORDD)
- Result Date (RSLT)
- Provider (PROV) – excluded from final model due to low performance

Development Steps:

1. Enumeration of relevant documents from the DataLake using filters on document type and description.
2. OCR conversion using AWS Textract [1][2].
3. Outlier detection using a custom implementation of OCNB (Outlier Classifier using 3-gram tokenization), a method aligned with recent advances in out-of-distribution detection in NLP [6].
4. Entity extraction using spaCy’s textcat pipeline [5].

5. Entity filtering based on Levenshtein distance and fuzzy matching thresholds, which are widely used in clinical text normalization [7].
6. Deduplication and normalization of entity spans.
7. Training using spaCy's optimizer with custom learning rate and gradient noise schedules.
8. Training parameters included 10 epochs, a learning rate schedule (eta=0.0005, gamma=0.2, t_0=1), and a gradient noise schedule (eta=0.2, gamma=0.4, t_0=1).

3. Training Data Source and Performance Metrics

Data used for model development represented inbound colonoscopy- and Cologuard-related documents received via fax scanned into the health system between 2010 and 2023 (Figures A1 & A2). The validation and training data sets were generated though a 4:1 split. The model was trained on a dataset of 7,021 colonoscopy reports and validated on a separate test set of 1,758 documents. These documents were sourced from OnBase and filtered to include only those with valid linked orders and relevant descriptors (e.g., containing "COLON" or "COLOGUARD").

Table A1. Performance Metrics (Test Set)

Entity	Precision (%)	Recall (%)	F1 Score (%)
DOB	97.18	98.12	97.65
First Name	95.88	95.26	95.57
Follow-Up Interval	97.64	99.90	98.76
Last Name	93.95	94.00	93.97
Order Date	90.63	83.81	87.09
Provider	74.97	77.26	76.10
Result Date	50.14	86.74	63.55

4. Internal Validation Strategy

Internal validation was performed using a patient- and time-based split, ensuring that training and test sets did not overlap in terms of patient records or time periods. This approach helps mitigate data leakage and improves generalizability [4].

5. Model Success Measures and Practical Costs of Errors

Success was defined by the model's ability to accurately extract entities and determine whether a report was "RPA Ready." This also represented the correct identification of an available follow-up date without identifying any false positive dates.

Errors in entity extraction—particularly for dates and names—could result in incorrect or delayed data entry into patient records, potentially impacting clinical workflows. Potentially incorrect follow-up frequency documented into health maintenance (HM) activity. The model prioritizes high precision and recall for critical fields such as DOB and Follow-Up Interval to minimize such risks.

6. Data Characteristics and Generalizability

The training data included a diverse set of scanned reports from multiple institutions and represented the results of both colonoscopy and Cologuard reports. 91% of documents included in the data set were colonoscopy reports and 6% were pathology reports (Table A2). Data was received by 267 different NYULH sites. Due to meta-data being stripped at the time of fax scanning original document sources (i.e. sender) could not be obtained. Documents varied in format, length (50–5000 characters), and OCR confidence scores (minimum average word confidence of 70%). Entity lengths were capped at 25 characters to reduce noise.

Table A2. Document type in training and validation data sets.

**Document Type**	**Overall**	**Training Set**	**Validation Set**
TOTAL	8779	7021	1758
Colonoscopy	7955	6358	1597
Pathology	587	473	114
Outside Record	78	62	16
Consult Letters	42	32	10
Other	33	25	9
External Documents	20	17	3
Radiology Result	19	18	1
CT Scan	8	4	4
Endoscopy	7	6	1
Lab Result	9	7	2
Diagnostic Report	5	5	0
Correspondence	4	4	0
Outside Order	3	2	1
Operative Report	2	2	0
Prescriptions	1	1	0
Biopsy Req	1	1	0
Historical Logician Document	1	1	0
Consent Form	1	1	0
EKG	1	1	0
Discharge Instruction	1	1	0
Procedure Note	1	1	0

Figure A1. Model training data by source scanned date

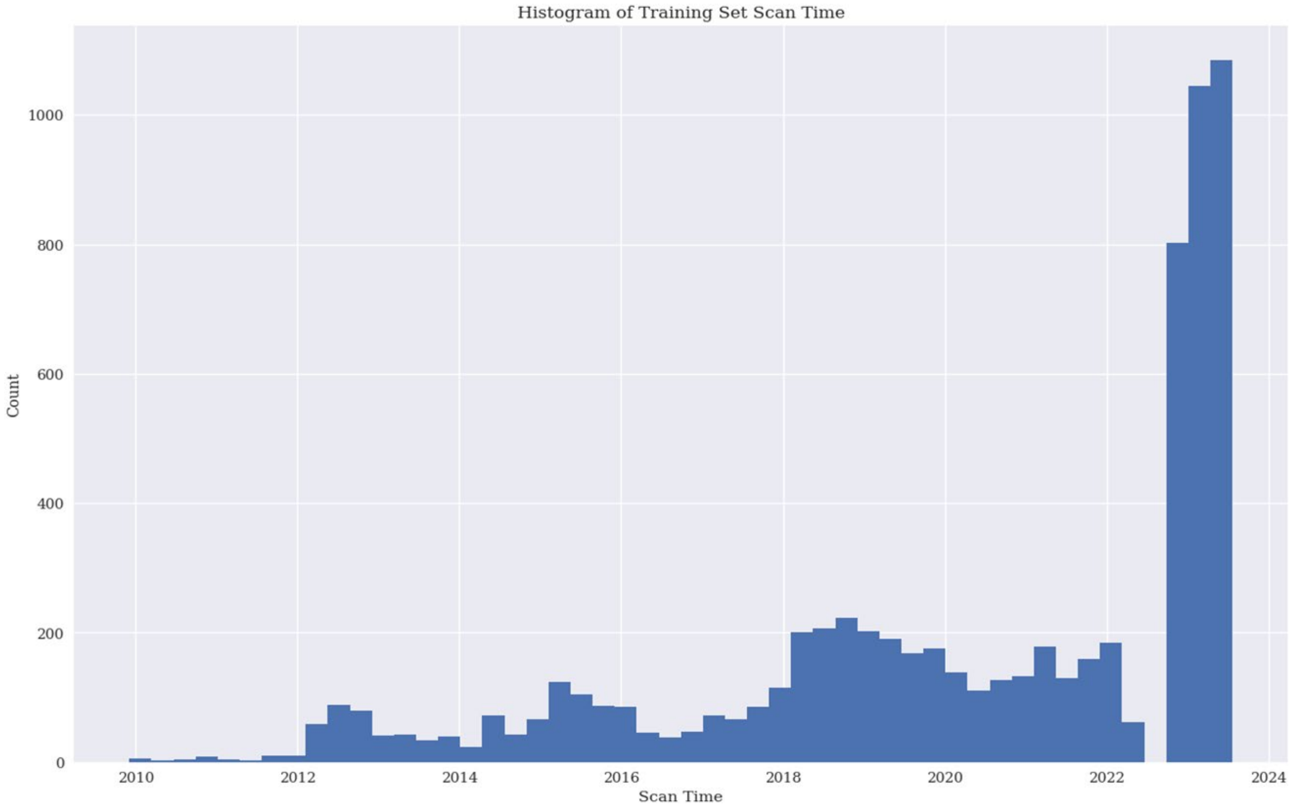

Figure A2. Model validation data by source scanned date

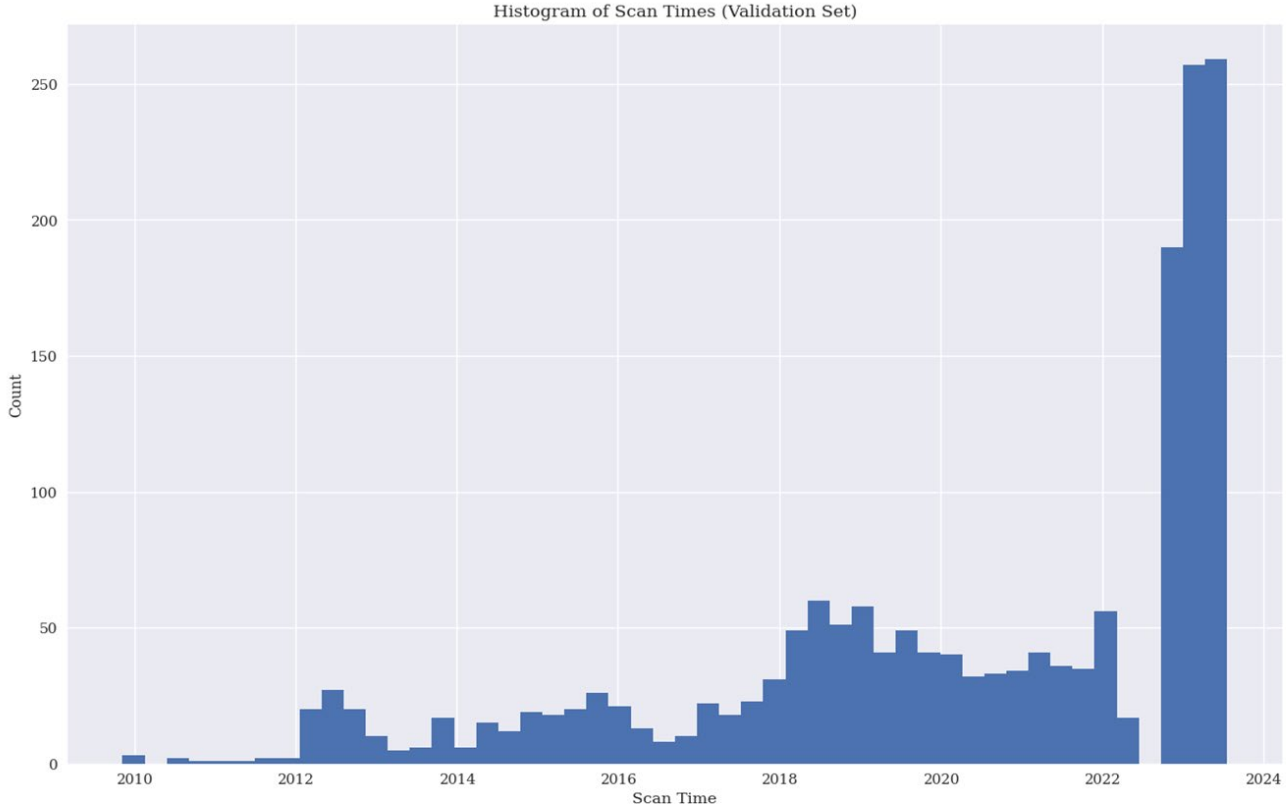

7. **Model code**
    - NER model built on spaCy v2.3.2
    - AWS Textract for OCR of image to get raw text.
    - Custom OCNB implementation for outlier detection using 3-gram tokenization.
    - regex Python package (different from re) and fuzzywuzzy (Levenshtein Distance calculation) for fuzzy partial matching on entities.
- What variables were in the model? - spaCy textcat pipe
    - First Name (FN)
    - Last Name (LN)
    - Date of Birth (DOB)
    - Follow Up Interval (FOLUP)
    - Order Date (ORDD)
    - Result Date (RSLT)
    - Provider (PROV) - **Not Used**
- What proportion of various input sources?
    - Need to run another report to get this info though we tried to pull all relevant documents from OnBase at the time.

Model Inference (input / output):

1. **Input:** Weekly extract of OnBase scans matching DOC_TYPE='20004' (Colonoscopy)
2. DOC_LINKED_ORDERS pulled to line up patient / order info to compare with extracted entities.
3. Raw text is generated using AWS Textract API.
4. spaCy NLP extracts valid entities that are then checked against patient info.
5. Entities de-duped based on maximum fuzzy partial ratio score to matched info and valid date formats.
6. Composite score generated using fuzzy partial ratios to determine if record is "RPA Ready" or "RPA Not Ready".
7. **Output:** Excel sheet generated to hand off to RPA for upload into patient record.

Training Parameters:

Training Workflow

1. Relevant documents enumerated from DataLake in a report with valid entities
    - DOC_TYPE=200004 (Colonoscopy)
    - DOC_DESCR contains '%COLON%' or '%COLOGUARD%'
    - Documents from OnBase (filtered out blobstore docs).
    - DOC_LINKED_ORDER exists / scanned to order (instead of scanning to patient / encounter).
2. OnBase pulls all documents from enum.
3. Documents processed through AWS Textract to get raw text.
4. OCNB (unsupervised) trained on raw text for outlier detection.
5. Documents and entities filtered by below specification.
6. Entities de-duped and spans are normalized.
7. spaCy textcat pipeline trained using the spaCy optimizer with parameters:
    - Gradient Noise schedule based on (eta=0.2, gamma=0.4, t_0=1): https://arxiv.org/abs/1511.06807
    - Learning Rate schedule based on (eta=0.0005, gamma=0.2, t_0=1): https://arxiv.org/pdf/1803.02865.pdf
    - epochs = 10

- Train Set: 7021
- Test Set: 1758

Label Filter Parameters

Document Filter Params
min_chars = 50
max_chars = 5000
min_words = 10
max_words = 600
min_word_conf = 70 # Average AWS Textract WORD block confidence

Entity Filter Params
Based on Levenshtein distance:
* Substitutions <= 1
* Insertions <= 1
* Deletions <= 1
* Total Errors <= 3
* Fuzzy Partial Ration >= 80 (60 for datetime entities)

max_ent_length = 25 (filter out entities longer than 25 characters long).

Test Set Performances

TEST:

ENT	P	R	F
DOB	97.184	98.117	97.648
FN	95.882	95.256	95.568
FOLUP	97.640	99.899	98.757
LN	93.948	93.996	93.972
ORDD	90.628	83.813	87.088
PROV	74.970	77.262	76.099
RSLT	50.144	86.740	63.550

8. References

[1] Amazon Web Services. Textract. https://aws.amazon.com/textract/
[2] Rajpurkar, P., et al. (2018). 'Deep learning for chest radiograph diagnosis: A retrospective comparison of the CheXNeXt algorithm to practicing radiologists.' PLoS medicine.
[3] Levenshtein, V. I. (1966). 'Binary codes capable of correcting deletions, insertions, and reversals.' Soviet physics doklady.
[4] Luo, Y., et al. (2016). 'Guidelines for Developing and Reporting Machine Learning Predictive Models in Biomedical Research.' Journal of Biomedical Informatics.
[5] spaCy Documentation. https://spacy.io/
[6] Hendrycks, D., & Gimpel, K. (2017). 'A baseline for detecting misclassified and out-of-distribution examples in neural networks.' arXiv preprint arXiv:1610.02136.
[7] Sarker, A., & Gonzalez, G. (2015). 'Portable automatic text classification for adverse drug reaction detection via multi-corpus training.' Journal of biomedical informatics.
